# Supplementary material for: The interactome of intact mitochondria by cross-linking mass spectrometry provides evidence for coexisting respiratory supercomplexes
Source: Mol Cell Proteomics. 2017 Dec 8;17(2):216–32. doi: 10.1074/mcp.RA117.000470 (PMC5795388; doi:10.1074/mcp.RA117.000470)
Supplement: Supplemental Data [file supp_RA117.000470_133922_0_supp_23978_fzffwf.zip › spectra_annotation/mito_spectra_annotation/125-1-3-1-12-1.pdf]

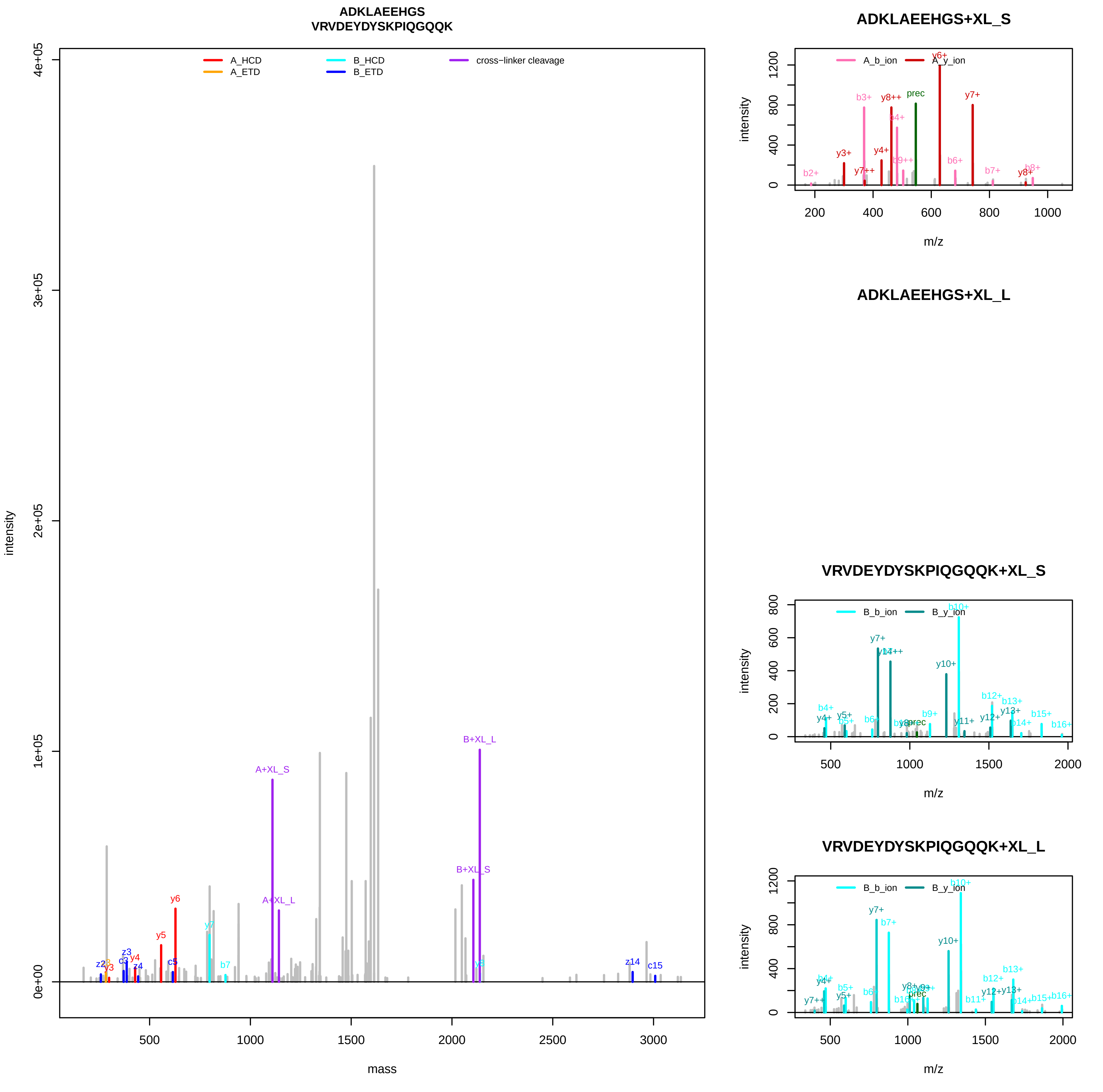
ADKLAEEHGS
VRVDEYDYSKPIQGQQK
A_HCD
A_ETD
B_HCD
B_ETD
cross−linker cleavage
intensity
mass
0e+00
1e+05
2e+05
3e+05
4e+05
500
1000
1500
2000
2500
3000
z2
c3
z3
y3
y4
z4
y5
y6
c5
y7
b7
A+XL_S
A+XL_L
B+XL_L
B+XL_S
z14
c15
ADKLAEEHGS+XL_S
A_b_ion
A_y_ion
b2+
y3+
y7++
b3+
y8++
b4+
y4+
b9++
prec
y6+
b6+
y7+
b7+
y8+
b8+
m/z
ADKLAEEHGS+XL_L
VRVDEYDYSKPIQGQQK+XL_S
B_b_ion
B_y_ion
b4+
y4+
y5+
b5+
y7+
y14++
b6+
y8
prec
b9+
y10+
b10+
y11+
b12+
y12+
b13+
y13+
b14+
b15+
b16+
VRVDEYDYSKPIQGQQK+XL_L
y7++
y5+
b5+
b7+
y8+
y9+
b16+
y10+
b11+
y12+
y13+
b14+
b15+
